# Hysteresis in memristors produces conduction inductance and conduction capacitance effects

**DOI:** 10.1039/d4cp00586d

**Published:** 2024-04-15

**Authors:** Juan Bisquert, Juan B. Roldán, Enrique Miranda

**Affiliations:** a Institute of Advanced Materials (INAM), Universitat Jaume I 12006 Castelló Spain bisquert@uji.es; b Departamento de Electrónica y Tecnología de Computadores, Universidad de Granada, Facultad de Ciencias Avd. Fuentenueva s/n 18071 Granada Spain; c Dept. Enginyeria Electrònica, Universitat Autònoma de Barcelona 08193 Cerdanyola del Vallès Spain

## Abstract

Memristors are devices in which the conductance state can be alternately switched between a high and a low value by means of a voltage scan. In general, systems involving a chemical inductor mechanism as solar cells, asymmetric nanopores in electrochemical cells, transistors, and solid state memristive devices, exhibit a current increase and decrease over time that generates hysteresis. By performing small signal ac impedance spectroscopy, we show that memristors, or any other system with hysteresis relying on the conductance modulation effect, display intrinsic dynamic inductor-like and capacitance-like behaviours in specific input voltage ranges. Both the conduction inductance and the conduction capacitance originate in the same delayed conduction process linked to the memristor dynamics and not in electromagnetic or polarization effects. A simple memristor model reproduces the main features of the transition from capacitive to inductive impedance spectroscopy spectra, which causes a nonzero crossing of current–voltage curves.

## Abstract 2

Memristors display hysteresis effects in the form of self-crossing looping current–voltage curves. We show intrinsic dynamic inductor-like (conduction inductance) and capacitance-like (conduction capacitance) behaviours.

## Graphical abstract

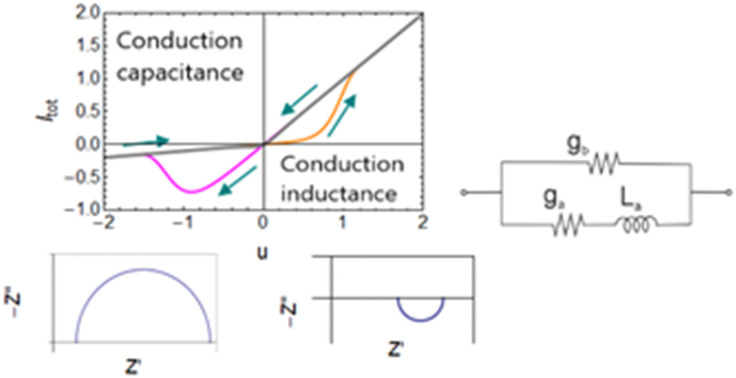

Memristive devices are being widely investigated for different applications related to non-volatile memory storage, neuromorphic computational systems, hardware cryptography and radio-frequency switches.^1–8^ Memristors are often defined by equations of the type^9–13^

1 *I*_tot_ = *f*(*V*, *λ*)

2
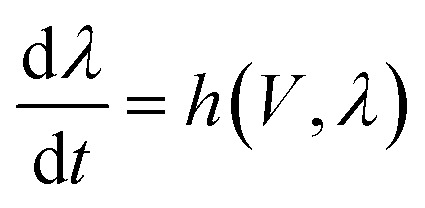


Eqn (1) is a current–voltage *I*_tot_–*V* characteristic that depends on a state variable *λ*. This variable imparts memory characteristics to the system *via* relaxation dynamical equation eqn (2). The adaptation of *λ* is delayed with respect to the changes of the stimulus of the external voltage. By the delay effect (incremental form) of eqn (2), memristors always show hysteresis.

Memristors, as described by eqn ((1) and (2)), are usually associated with the properties of resistive switching,^13^ often connected with the formation and partial disruption of a nonvolatile conduction filament (in the most general case of filamentary switching) in the insulator layer that separates metal electrodes. However, a larger class of systems are also described by these equations. For example, solar cells, protein channels, and transistors show hysteresis,^14–21^ while the resistance memory is not the main functionality, and are normally not associated with resistive switching. To provide a more general account of these properties, we have termed a chemical inductor as any conduction system described by eqn ((1) and (2)).^22^ The central paradigm of the chemical inductor, and historically the original formulation, is given by the Hodgkin–Huxley equations that describe the temporal dynamics of action potentials in neurons.^23–26^

Memristors are usually characterized by a self-crossing current–voltage curve under a dynamic scan of voltage. This is because the current raises in one polarity, *e.g.* at a positive voltage, which is called a SET process, and in the return RESET process at negative polarity, the current returns to a low value. An example of the experimental measurement of rectifying nanopores in electrochemical cells^18,27,28^ is shown in Fig. 1.^29^

**Fig. 1:**
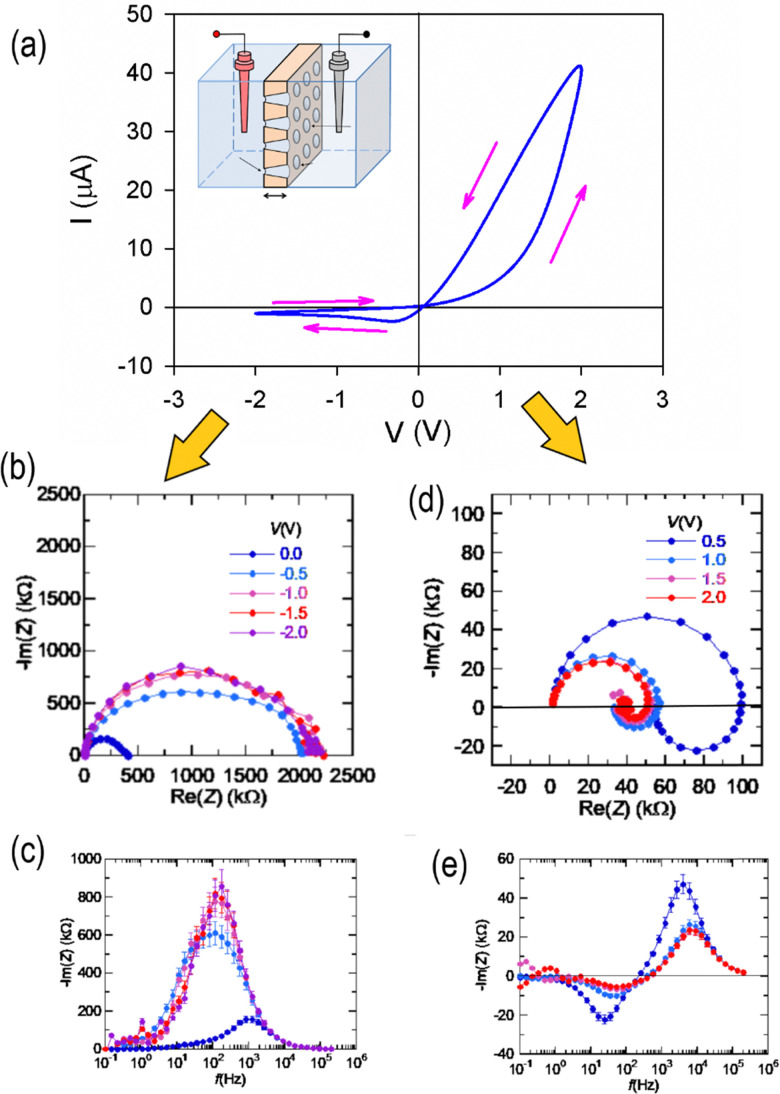
(a) Current–voltage curve measured at a frequency of *Ω*_s_ = 10 Hz for a multipore membrane in 100 mM KCl solution at a neutral pH. The inset shows the electrochemical cell with the membrane. (b) The impedance spectra at different reverse voltages, with the corresponding bode plot of the imaginary part of the impedance in (c). (d) and (e) The impedance spectra and bode plots at a forward voltage. Adapted from P. Ramirez, J. Cervera, S. Nasir, M. Ali, W. Ensinger and S. Mafe, Electrochemical impedance spectroscopy of membranes with nanofluidic conical pores, *J. Colloid and Interface Sci.*, 2024, **655**, 876–885, with permission from Elsevier.^29^

In this paper, we discuss the frequency domain response of memristors and self-crossing chemical inductors, in general, and we analyze the relationship of such properties with the hysteresis features. We show here that for a system described by eqn ((1) and (2)), there are certain regions of operation where one might interpret a measurement of a memristor to be a capacitor and in other regions to be an inductor. We emphasize that there is no real capacitor or inductor (*i.e.*, no sub-components with a Q–V or Flux–I relationships). Rather, a memristor behaves in “capacitor-like” and “inductor-like” manners. This result is obtained by representing the small signal ac impedance response of the memristor in the typical form of an equivalent circuit.^30–34^

It has been possible to classify the types of hysteresis displayed by memristors.^35^ In a set cycle, where the conductance changes to a high value, the *I*–*V* curve makes a counter clockwise loop. The hysteresis is inductive (or inverted), see Fig. 1a, for positive voltages.^36–42^ In contrast, in the reset cycle, the current decreases and the hysteresis is capacitive, making a clockwise loop, see Fig. 1a at negative voltages. Furthermore, it has been shown that the small signal ac impedance characteristics reveal the type of hysteresis inherent to the dynamical properties of the model.^35^ The inductive characteristic, represented by the arc in the fourth quadrant of the complex impedance plane related to the counter clockwise cycle of the current–voltage in the positive voltages, is reported in Fig. 1d. Contrarily, in the negative side, the impedance is fully capacitive and associated with the clockwise reset loop.

The inductive feature of impedance spectroscopy has been observed in different types of all-solid memristors.^42–46^Fig. 2 shows the huge inductive arcs of a halide perovskite memristor. This is the famous “negative capacitance” of solar cells,^47–50^ which has been connected to inverted hysteresis^36,37,51^ that here we also term inductive hysteresis.^35^ In Fig. 3, the conductance and susceptance of a TiN/Ti/HfO_2_/W resistive RAM memory device are shown.^46^ It is observed that when the conductance switches between low (OFF state) and high (ON state), the susceptance changes respectively between positive (capacitive) and negative (inductive).

**Fig. 2:**
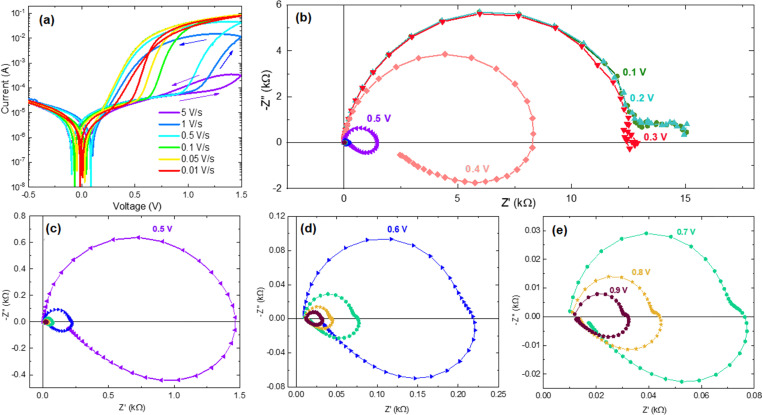
(a) Current–voltage characteristics of the halide perovskite memristor device FTO/poly(3,4-ethylenedioxythiophene) polystyrene sulfonate (PEDOT:PSS)/CH_3_NH_3_PbI_3_/Au at 6 different scan rates starting from 5 V s^−1^. Arrows indicate the sweep direction. (b)–(e) Complex plane plot representation of the impedance spectra at different applied dc voltages. (c)–(e) Plots corresponding to a magnification of the scales. M. Berruet, J. C.Pérez-Martínez, B. Romero, C. Gonzales, A. M. Al-Mayouf, A. Guerrero and J. Bisquert, Physical model for the current–voltage hysteresis and impedance of halide perovskite memristors. *ACS Energy Lett.*, 2022, **7**, 1214–1222; licensed under a Creative Commons Attribution (CC BY) license.^43^

**Fig. 3:**
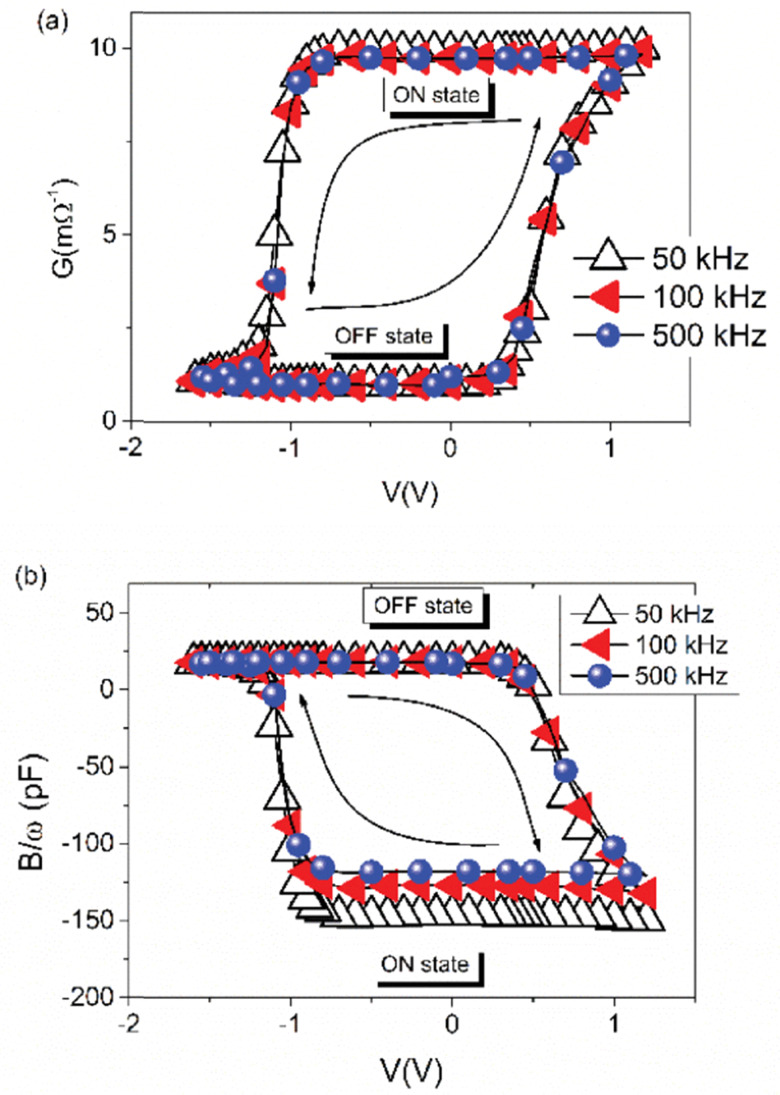
Conductance (a) and susceptance (b) of the TiN/Ti/HfO_2_/W resistive RAM memory device measured at 0 volts *vs.* programming voltage at three frequencies. An increasing and decreasing voltage steps applied in the range in which the device switches. Between the steps, the impedance is measured at zero voltage, applying only the small ac signal to avoid introducing disturbance. Reproduced by permission from S. Dueñas, H. Castán, H. García, Ó. G. Ossorio, L. A. Domínguez, and E. Miranda, Experimental Observation of Negative Susceptance in HfO_2_-Based RRAM Devices, *IEEE Electron Device Lett*., 2017, **38**, 1216–1219.^46^

The appearance of a large inductor in systems that do not contain any internal electromagnetic inductor has been remarked in many research fields, *e.g.* in solar cells,^47–50^ corrosion systems,^52,53^ and proton exchange membrane (PEM) fuel cells.^54–56^ Since the early part of the 20th century, people have modeled many systems (*e.g.*, biological squid axons) and noticed that they exhibit anomalously large inductance. The inductive behaviour of the non-magnetic origin in the squid giant axon was well recognized before 1940 by Cole, based on impedance spectroscopy measurements,^57^ but he remarked that “The suggestion of an inductive reactance anywhere in the system was shocking to the point of being unbelievable.”^58^ Hodgkin and Huxley^23^ proposed that the potassium conductance is proportional to a power of a variable that obeys a first-order equation, in order to match the very different transient curves: the delayed increase in depolarization, but a simple exponential decay in repolarization. Thus, as explained later by Hodgkin,^59^ “the inductance is mainly due to the delayed increase in potassium conductance which can make the membrane current lag behind the voltage provided that the internal potential is positive to the potassium equilibrium potential.” The inductive response of the neuron model has been explained by Chua, based on eqn ((1) and (2)).^60,61^ A variety of models with negative chemical inductors have also been described.^62^

Recently, we have described in a general fashion the impedance properties exhibited by chemical inductors^22,62^ including neurons^63,64^ and memristors.^43,65^ As shown in these works, from eqn ((1) and (2)), it follows that the inductive-like behaviour is an inherent property to the system (with no electromagnetic induction present), what justifies the name of the “chemical inductor”, in reference to the dynamical response of the system and not to its specific physical or chemical mechanism. In practice, a wide variety of effects are described in this way and generate an inductor, *e.g.*, the inertia of electrons in the Drude model.^66^ There are many processes that can potentially cause the inductive delay, like ionic motion in perovskite single crystals,^42^ and sometimes they are not known in advance. However, the general feature of these systems is that the inductor relates to a delayed conduction process.

In contrast to these general remarks, the capacitive response observed in the reset cycle has not been highlighted enough. The capacitance is an ubiquitous property in any real conducting system. In previous studies, we included in eqn (1) a capacitive current3
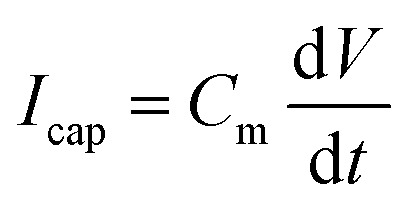
which is always present, *e.g.*, the neuron wall capacitance in the Hodgkin–Huxley model,^23–26^ or the geometric capacitance in semiconductors or dielectric devices. However, a constant capacitance related to bulk or surface polarization effects responds independently of the applied voltage, and the response current depends only on the signal rate, eqn (3). In fact, *C*_m_ cannot produce the capacitive cycles (*e.g.*, clockwise in Fig. 1) that are observed in all systems with hysteresis, when the low conductance state is recovered. In some systems, the state variable of the memristor produces a polarization component as well, causing a variable capacitive current d*λ*/d*t*.^67^ However, this mechanism is far from being universal.

Therefore, one may wonder which is the origin of the measured capacitance obtained in the reversal of memristors to the low conductance state?

In this paper, we investigate this question in detail. We take the basic behavioral memristor model developed by Miranda *et al.*^68–70^ which makes no reference to any geometric capacitance and we show how the conduction capacitance associated with the reset cycle naturally emerges.

The model^68^ is formulated through the equations:

4 *V* = *R*_s_*I*_tot_ + *u*

5 *I*_tot_ = [*g*_L_ + (*g*_H_ − *g*_L_)*λ*]*u*

6
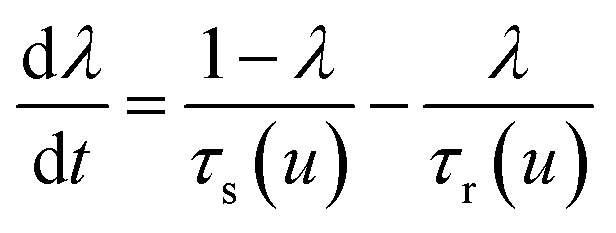
where *R*_s_ is an external series resistance, the variable *u* is the voltage inside the device, and *g*_L_ and *g*_H_ are conductances for the low and high conductance states of the memristor, respectively. The relaxation (characteristic) times for set and reset are given by:7
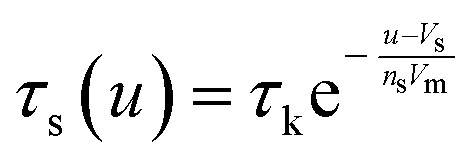
8
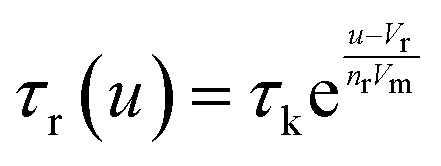
with parameters *τ*_k_, *V*_s_, *V*_r_, *V*_m_, *n*_s_, and *n*_r_.

The equilibrium condition is obtained from eqn (6)9
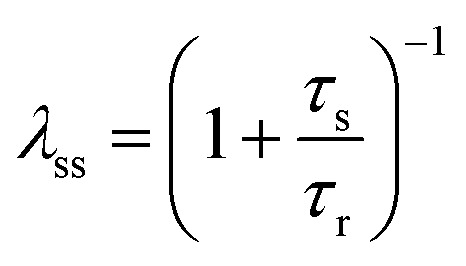
which can be explicitly expressed as follows:10
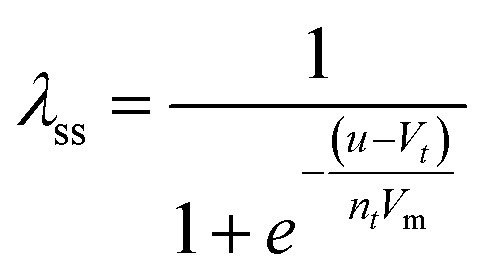


This function is activated at the voltage *V*_*t*_, defined by the equations11
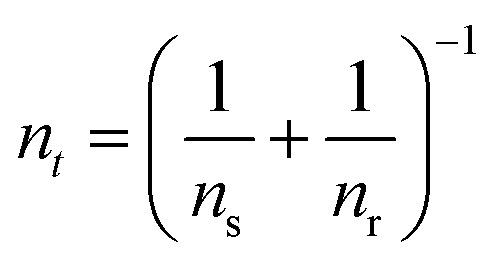
12
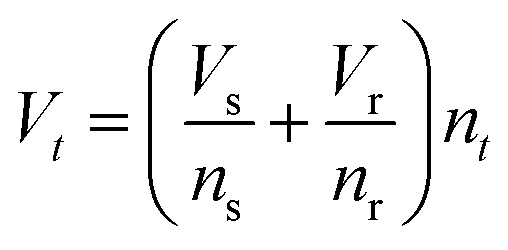


As shown in Fig. 4a, the current makes a transition from the low conductance *g*_L_ to *g*_H_ as the voltage changes. The activation process corresponding to the memory variable (change from 0 to 1) is shown in Fig. 4b. Other memristor models,^65,71^ make use of a sigmoidal activation function (10). This form corresponds to a Boltzmann open channel probability^72,73^ that indicates the fraction of active conducting channels according to the applied voltage.

**Fig. 4:**
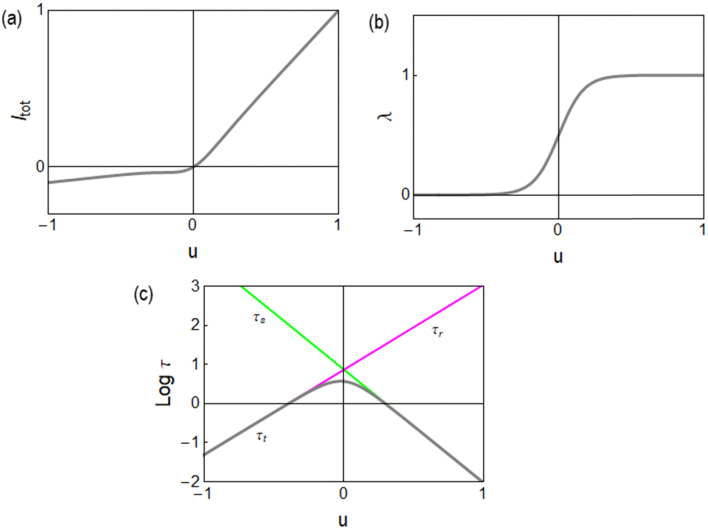
(a) Current–voltage curve. (b) Equilibrium value of the state variable. (c) Relaxation times. Parameters *V*_s_ = 0.3, *V*_r_ = −0.4, *V*_m_ = 0.1, *n*_s_ = 1.5, *n*_r_ = 2, *g*_L_ = 0.1, *g*_H_ = 1, and *τ*_k_ = 1.

Under a constant voltage sweep at rate *v*_scan_

13 *u* = *v*_scan_*t*

the relaxation properties produce the hysteretic behaviour shown in Fig. 5a, conducted by the out-of-equilibrium cycling of the memory variable *λ* shown in Fig. 5b. Clearly, the hysteresis is inductive at the positive voltage side, and capacitive at the negative side.^35^ It is observed that the model represents well the rectifying and hysteresis properties of asymmetrical nanopores as those shown in Fig. 1.

**Fig. 5:**
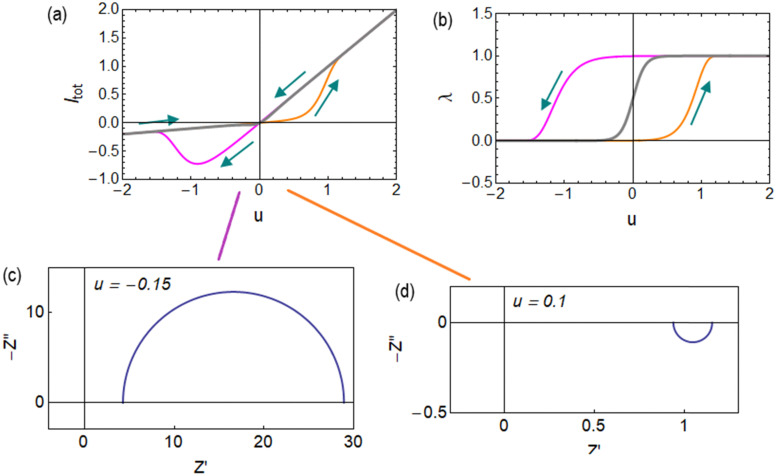
Hysteresis at a rate *v*_scan_ = 10 in (a) current–voltage curves and (b) state variable. The grey lines are the equilibrium curves. (c) and (d) Impedance spectra at the indicated voltage points. Parameters as in Fig. 4.

To obtain further insight into the dynamical properties of the model, we calculate the small signal ac impedance response at the angular frequency *ω*. As usual,^34,43^ the equations are expanded to the first order, where the perturbation of variable *y* is indicated as *ŷ*, and the factor functions of each term are computed under equilibrium conditions. Furthermore, we transform the small signal equations to the frequency domain by the Laplace transform, d/d*t* → *s*, where *s* = *iω*. We obtain the equations

14 *Î*_tot_ = [*g*_L_ + (*g*_H_ − *g*_L_)*λ*]*û* + (*g*_H_ − *g*_L_)*u

<svg xmlns="http://www.w3.org/2000/svg" version="1.0" width="11.333333pt" height="16.000000pt" viewBox="0 0 11.333333 16.000000" preserveAspectRatio="xMidYMid meet"><metadata>
Created by potrace 1.16, written by Peter Selinger 2001-2019
</metadata><g transform="translate(1.000000,15.000000) scale(0.011667,-0.011667)" fill="currentColor" stroke="none"><path d="M480 1160 l0 -40 -40 0 -40 0 0 -80 0 -80 40 0 40 0 0 80 0 80 40 0 40 0 0 -80 0 -80 40 0 40 0 0 80 0 80 -40 0 -40 0 0 40 0 40 -40 0 -40 0 0 -40z M400 840 l0 -40 -40 0 -40 0 0 -40 0 -40 40 0 40 0 0 40 0 40 40 0 40 0 0 -160 0 -160 -40 0 -40 0 0 -40 0 -40 -40 0 -40 0 0 -40 0 -40 -40 0 -40 0 0 -80 0 -80 -40 0 -40 0 0 -40 0 -40 -40 0 -40 0 0 -40 0 -40 40 0 40 0 0 40 0 40 40 0 40 0 0 40 0 40 40 0 40 0 0 80 0 80 40 0 40 0 0 40 0 40 40 0 40 0 0 -200 0 -200 80 0 80 0 0 40 0 40 40 0 40 0 0 40 0 40 -40 0 -40 0 0 -40 0 -40 -40 0 -40 0 0 360 0 360 -40 0 -40 0 0 40 0 40 -40 0 -40 0 0 -40z"/></g></svg>

*

15
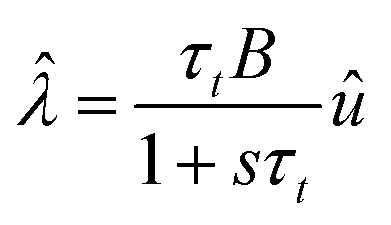
here we use the auxiliary functions16
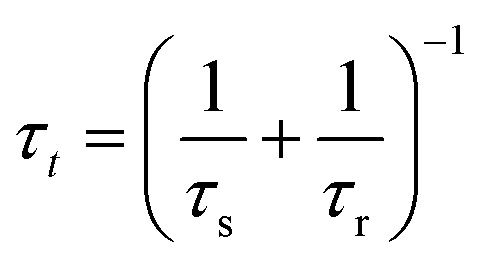
17
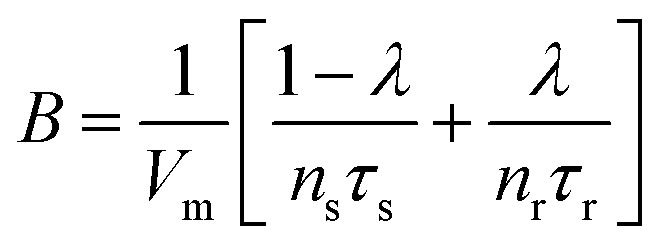


These functions are plotted in Fig. 4c and 6b. The solution of the impedance obtained from 14 and 15 is18
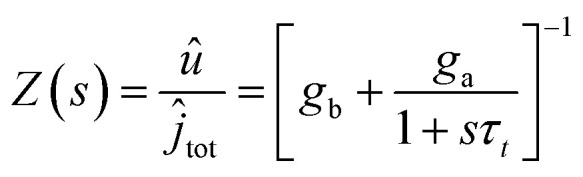


**Fig. 6:**
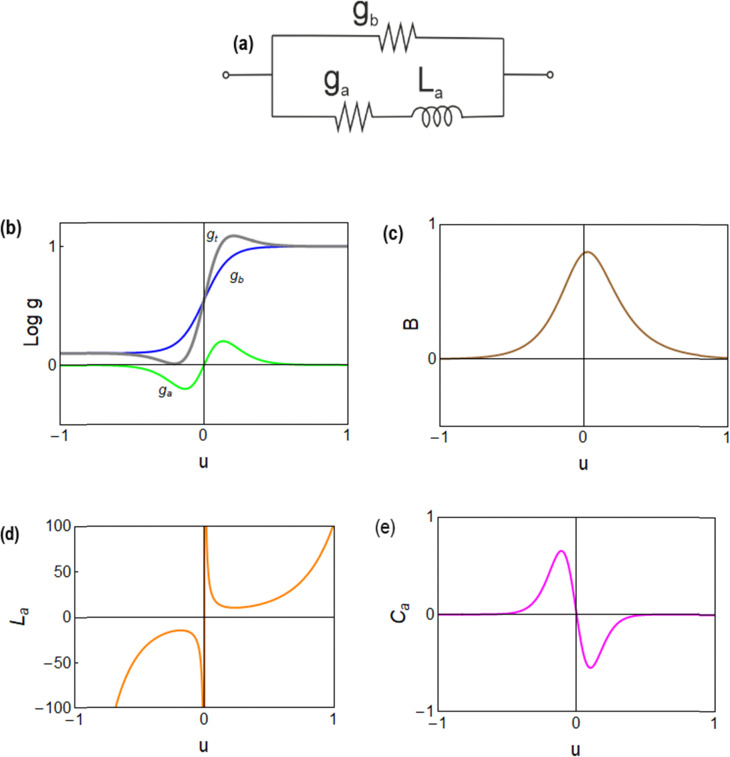
(a) Equivalent circuit. Functions determining the impedance response. (b) Differential conductances, *g*_*t*_ = *g*_b_ + *g*_a_. (c) *B*. (d) Inductor. (e) Effective low frequency capacitance. Parameters as in Fig. 4.

The equivalent circuit corresponding to eqn (18) is shown in Fig. 6a. In this case, the circuit elements are defined by the relationships

19 *g*_b_ = *g*_L_ + (*g*_H_ − *g*_L_)*λ*

20 *g*_a_ = *B*(*g*_H_ − *g*_L_)*τ*_*t*_*u*

21
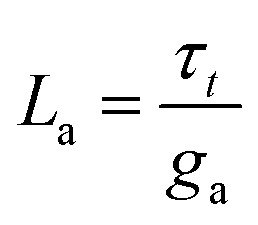


The behavior of differential conductance is shown in Fig. 6b. The resulting impedance spectra both at positive and negative potentials are shown in Fig. 5c and d. In the set side, the impedance shows an inductor feature, and as it has been explained in many publications, it is associated with the inverted (inductive) hysteresis.^22,35^ In the negative side, the impedance spectra are purely capacitive, as also observed in the experimental data of Fig. 1. However, the impedance model of eqn (18) and Fig. 6a contains solely an inductor but not a capacitor element. How is the capacitor generated at negative voltages?

To address this question, we calculate the effective capacitance corresponding to the branch (*R*_a_, *L*_a_) with impedance *Z*_a_ = *g*_a_^−1^ + *sL*_a_. This capacitance is given by the expression22
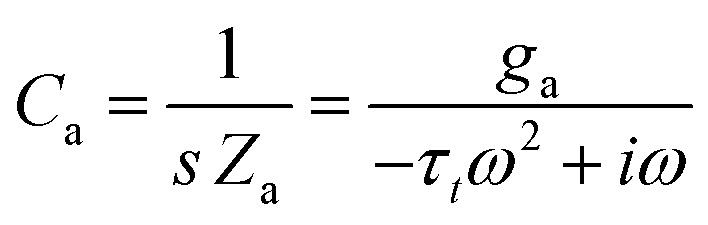
Therefore23
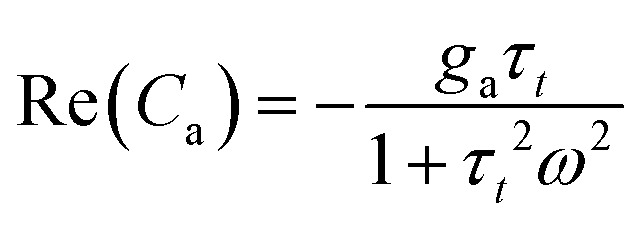


From (23), if *g*_a_ > 0, then the system generates the negative capacitance effect that characterizes the chemical inductor.^44,47,74–81^ However, for *g*_a_ < 0, the model produces a positive low frequency capacitance. We can see in Fig. 6b that the change of the sign of *g*_a_ occurs at *V* = 0, causing the positive capacitance at negative voltages as shown in Fig. 6e. We conclude that the capacitance observed in the reset process originates in the same delayed conduction process as the inductor in the set process.

It is also remarked that the inductor element *L*_*a*_ in Fig. 6d becomes negative when *g*_a_ is negative.^62^ This ensures that the small perturbation relaxation time *τ*_*t*_ = *g*_a_*L*_a_ is positive at all voltages (as required for a stable system^62^), as shown in Fig. 4c.

While the model described applies to a variety of systems with specific conduction and memristive mechanisms, it is worthwhile to discuss the expected value of the conduction inductor and conduction capacitance, which have produced doubts of interpretation when measured in some systems, as remarked before.^57^ Consider a conductance of 1 nA V^−1^ for a single pore^82^ or a memristor filament, a kinetic time *τ*_*k*_ is 1 s. The value of the inductor can be estimated as24
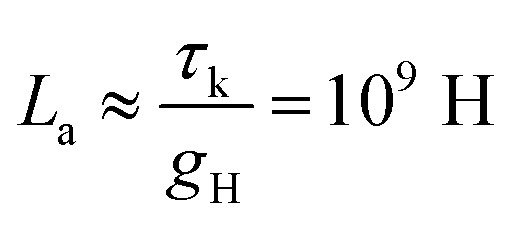


Even if there are 100 conducting units per cm^2^, as shown in Fig. 1, the conduction inductance can reach a very large value of 10^7^ H cm^−2^. Obviously, for systems with a short relaxation time, the inductor will be smaller, in the order of μH.^46,83^ For the conduction capacitance of a single unit in the same example

25 *C*_a_ = *g*_H_*τ*_k_ = 1 nF

The conduction capacitance is higher than typical geometric capacitances, but smaller than chemical capacitances.^84^

In summary, observing large values of inductance and capacitance is good evidence that a memristor has been measured and not an *L* or a *C*.

It should be noticed that the use of (7) and (8) predict finite switching times a zero voltage. If required, they can be replaced by expressions in which the switching times become infinite (d*λ*/d*t* → 0) at zero voltages. These modifications have been suggested in recent publications;^69,70^ however, the general mechanism based on eqn (6) for the generation of both capacitance and induction in the ac response should be the same, although with more complex analytical expressions.

The model is very useful to explain other phenomena that have been identified in the research of high-performance halide perovskite solar cells. This is the transition from capacitive to inductive hysteresis, which complicates the assessment of the power conversion efficiency.^36,39,85^ In this transformation, when the bias voltage increases, the low frequency capacitance becomes an inductor, with the associated transformation of capacitive to inductive hysteresis. This is illustrated in Fig. 7, and the same phenomenon is observed in Fig. 2. The transition has also been remarked in HfO_2_ resistive RAM memories, as shown in Fig. 3.^46^ The transition from capacitive to inductive or *vice versa* as the voltage increases produces a non-zero crossing of the *IV* curves, which has been recognized well in memristive systems.^66,86–88^

**Fig. 7:**
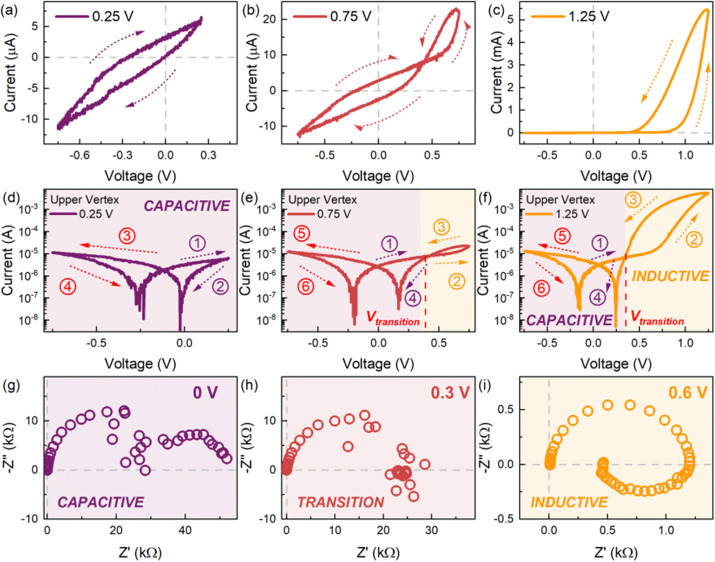
A. Characteristic current–voltage (*I*–*V*) response in the linear scale of the halide perovskite memristor device FTO/poly(3,4-ethylenedioxythiophene) polystyrene sulfonate (PEDOT:PSS)/CH_3_NH_3_PbBr_3_/Au with varying upper vertex voltages of (a) 0.25 V, (b) 0.75 V, and (c), 1.25 V, with the arrows indicating the scan direction. The corresponding *I*–*V* response in the semi-log scale for upper vertex voltages of (d) 0.25 V, (e) 0.75, and (f) 1.25 V with the arrows and numbers indicating the scan direction and sequence, respectively. Voltage-dependent impedance spectral evolution measured at (g) 0 V, (h) 0.3 V, and (i) 0.6 V exhibiting a transition from a low frequency capacitive response at low applied voltages to a low frequency inductive response at high applied voltages. Figure courtesy of Cedric Gonzales. Reproduced by permission from J. Bisquert, Inductive and capacitive hysteresis of current–voltage curves. A unified structural dynamics in solar energy devices, memristors, ionic transistors and bioelectronics., *PRX Energy*, 2023, **3**, 011001,^35^ licensed under a Creative Commons Attribution (CC BY 4.0) license.

To analyze the typical evolution of spectra, we add the constant capacitor (3) to the impedance model (18), so that the equivalent circuit of Fig. 8a is obtained. Now, the spectra display two capacitive arcs in the negative voltages, Fig. 8b. The high frequency arc close to the origin corresponds to the geometric capacitance, and the low frequency positive arc corresponds to the conduction capacitance. When approaching the transition voltage, the low frequency arc disappears and becomes an inductor, Fig. 8c, *i.e.* a negative arc. This is the experimental trend observed in Fig. 1, 2 and 7. This effect has been previously described using different models where the low frequency capacitance is coupled to the memory variable,^39,67^ as mentioned before. The model of Fig. 8a, based on eqn (6),^68^ explains well the general tendency of the transformation, with simpler physical components than those considered in previous approaches.

**Fig. 8:**
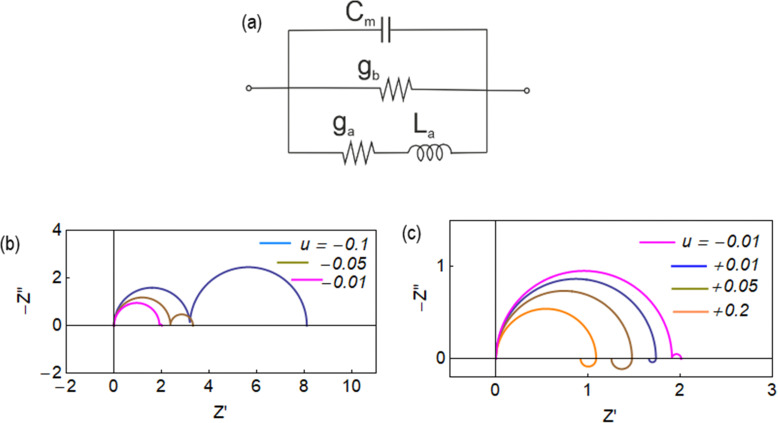
(a) Equivalent circuits and (b) and (c) impedance spectra at different stationary voltages. Same parameters as in Fig. 4 and *C*_m_ = 0.001.

In summary, a memristor is described by a set process in which the memory variable *λ* makes a transition 0 → 1 that promotes the increase of conductance. The associated impedance response is a conduction inductor that becomes quite large in some memristors.^43^ In the reset cycle, the opposite transition 1 → 0 takes place. We have shown that the impedance feature is a conduction capacitance in this range, and evolves to an inductor in the set range, explaining the transition from capacitive to inductive hysteresis that has been observed in different types of devices. We conjecture that this is a general property of systems with self-crossing hysteresis loops.

## Data availability

The data presented here can be accessed at https://doi.org/10.5281/zenodo.10952363 (Zenodo) under the license CC-BY-4.0 (Creative Commons Attribution-ShareAlike 4.0 International).

## Conflicts of interest

There are no conflicts to declare.

## Supplementary Material
